# Examining differences in menstrual and intimate care product use by race/ethnicity and education among menstruating individuals

**DOI:** 10.3389/frph.2023.1286920

**Published:** 2023-12-06

**Authors:** Ami R. Zota, Elissia T. Franklin, Emily B. Weaver, Bhavna Shamasunder, Astrid Williams, Eva L. Siegel, Robin E. Dodson

**Affiliations:** ^1^ Department of Environmental Health Sciences, Mailman School of Public Health, Columbia University, New York, NY, United States; ^2^ Silent Spring Institute, Newton, MA, United States; ^3^ Departments of Urban and Environmental Policy and Public Health, Occidental College, Los Angeles, CA, United States; ^4^ Black Women for Wellness, Los Angeles, CA, United States

**Keywords:** personal care products, health disparities, women's health, endocrine disruptors, feminine care, feminine hygiene, chemical exposures, fragrance

## Abstract

**Introduction:**

United States consumers spend over two billion dollars a year on intimate care products. These products, along with scented menstrual products, are marketed for odor control, perceived “freshness,” and vaginal/vulvar cleanliness. However, these scent-altering products may increase exposure to carcinogenic and endocrine-disrupting chemicals. Prior research has not adequately characterized demographic differences in product use. The objective of our study is to examine racial/ethnic and educational differences in menstrual and intimate care product use among people who menstruate.

**Methods:**

We pooled data from two US-based cross sectional studies to examine demographic characteristics and product use in 661 participants aged 18–54 years. Participants reported use of scented and unscented menstrual products (tampons, sanitary pads, and menstrual cups) and intimate care products (vaginal douches, sprays, wipes, and powders). We examined differences by race/ethnicity and education using log-binomial regression and latent class analysis (LCA), which can identify groups based on product use patterns.

**Results:**

Our sample was 33.4% Black, 30.9% Latina, 18.2% White, and 16.2% another identity. Approximately half the population had a bachelor's degree or more; 1.4% identified as transgender and 1.8% as non-binary. In adjusted models, scent-altering products (i.e., scented menstrual and intimate care products) were more likely to be used by those with less formal education (*p* < 0.05). Unscented menstrual products were more likely to be used by those with more formal education. Compared to Black participants, White participants were more likely to use unscented tampons and menstrual cups and less likely to use douches and wipes (*p* < 0.05). Using LCA we identified two groups: one more likely to use scent-altering products, and a second more likely to use unscented menstrual products. Less education and older age, but not race/ethnicity, was significantly associated with membership in the group more likely to use scent-altering products. While sex/gender composition did not statistically vary across groups, all non-binary participants fell in the unscented menstrual product group.

**Discussion:**

Lower educational attainment was consistently associated with greater use of scent-altering menstrual and intimate care products. Future research should examine associations between body odor stigma, product use, and health risks at intersections of race, class, and gender.

## Introduction

The feminine hygiene industry has been shaped by social, economic, and historical forces, which continue to impact contemporary product use. In the early to mid-20th century, there was rapid expansion in the commercial market for menstrual management. As indoor plumbing and disposable menstrual products became widely available, social expectations of bodily hygiene shifted to encourage the use of products that were marketed for odor control (1). Through curated advertisements that centered White, wealthy, and educated women, product manufacturers linked these “hygiene norms” with social mobility and privilege. The fear of stigma from body odor, and consequent menstrual and intimate care product use, was heightened after World War II when women entered male-dominated occupational settings (2). Adherence to these socially constructed “hygiene norms” was perceived as crucial to gaining access to professional opportunities, particularly for marginalized populations.

Today, sales of menstrual and intimate care products in the US are estimated at $3 billion dollars annually (3), and the global market is anticipated to reach $60 billion by 2030 (4). Yet, personal care products marketed for use near vaginal and vulvar tissues remain an understudied risk factor for reproductive health. Products of concern include: (1) menstrual products (e.g., tampons, sanitary pads, menstrual cups), which are used to manage menstrual bleeding, and (2) intimate care products (e.g., douches, vulvar sprays, wipes, powders), which are marketed for odor control and to help users attain perceived vaginal/vulvar cleanliness and freshness (2, 5, 6). Additionally, scented tampons and pads are marketed for both menstrual bleeding management and odor control. While product manufacturers commonly refer to menstrual and intimate care products as “feminine hygiene products” or “feminine care products,” we choose to use language that is inclusive of all menstruators regardless of gender identity. Additionally, we choose not to use the word “hygiene” to describe this product category since many of these products are marketed to medicalize normal bodily functions and create unnecessary concerns about cleanliness.

Menstrual and intimate care product use is relevant to population health because these products may contain one or more ingredients associated with allergies, asthma, cancer, endocrine disruption, and/or poor pregnancy outcomes. Table 1 summarizes the intended use and ingredients of concern of common products. There are now multiple studies that have quantified chemicals of concern in these products including asbestos, dioxins, per- and poly-fluoroalkyl substances, phthalates, parabens, metals, pesticides, volatile organic compounds (VOCs), and fragrance chemicals (e.g., alpha-isomethyl ionone, benzyl salicylate, hexyl cinnamaldehyde, linalool and piperonal) (13, 14, 22, 28, 34). Moreover, these products may represent an important source of human chemical exposure because they are used on highly permeable tissues that have high uptake rates and sensitivity to chemicals (34). Early data suggest that products marketed for odor control may be of particular concern. For example, scented tampons have higher concentrations of certain VOCs than unscented tampons, and the amount of fragrance chemicals leached from scented tampons has been demonstrated to exceed health protective thresholds for allergic reactions/skin sensitization (29, 35). Another study found that estimated cancer risks from VOCs exceeded health protective reference levels for sprays, washes, and powders (16).

**Table 1:** Overview of menstrual and intimate care products: product type, category, indicated reasons of for use, and chemicals of concern reported in product types.

	Category	Product description	Chemicals of concern
Tampons	Menstrual	Inserted into the vagina to collect menstrual fluids	Parabens (7)
			Triclosan (7)
			Dioxins & Furans (8–11)
			Pesticides (12)
			PAHs^a^ (11)
			Phthalates (7, 13, 14)
			Metals (15)
			VOCs^b^ (16, 17)
			Fragrances (14, 18)
Pads	Menstrual	Placed on underwear to collect menstrual fluids and other vaginal secretions	Parabens (8, 14)
			Chlorine (19)
			Triclosan (7)
			Dioxins & Furans (10, 20, 21)
			Biocides (11, 20)
			PAHs (11, 20)
			Phthalates (1, 5, 7, 11, 13, 14, 17, 22–27)
			Fragrances (11, 14, 18)
			VOCs (16, 28, 29)
Menstrual cups	Menstrual	Inserted into the vagina to collect menstrual fluids	VOCs (30)
			Phthalates (30)
			PAHs (30)
			PFAS^c^ (31)
Douches	Intimate care	Inserted into the vagina or anus to cleanse and prevent odor	Phthalates (32)
			VOCs (22)
			Fragrances (32)
Sprays	Intimate care	Sprayed onto genitals or underwear to reduce odor	VOCs (16)
			Phthalates (13)
			Parabens (13)
			Fragrances (18)
Powders	Intimate care	Sprinkled onto genitals, underwear, or menstrual products to absorb moisture and reduce odor	Talc (33)
			VOCs (13)
			Phthalates (27)
			Parabens (13) 11/2/23 2:20:00 PM
			Fragrances (18)
			Asbestos (33)
Wipes	Intimate care	Wiped on genitals or anus freshen up or removes odor	VOCs (14)
			Phthalates (13)
			Parabens (13)
			Ethanolamines (14)
			Fragrances (14, 18)

^a^ Polycyclic aromatic hydrocarbon.

^b^ Volatile Organic Compounds.

^c^ Per- and polyfluoroalkyl substances.

Two separate analyses of nationally representative National Health and Nutrition Examination Survey (NHANES) data found that the practice of vaginal douching was associated with increased exposure to certain phthalates and VOCs, with evidence of positive dose-response (e.g., higher biomonitoring levels among those who douche more frequently) (22, 36). Epidemiologic studies suggest that douching may be associated with pelvic inflammatory disease (37), ectopic pregnancy (23), bacterial and fungal vaginosis (24, 25, 38), and ovarian cancer (39), and genital use of talc-based powders may be a risk factor for ovarian cancer (40). Furthermore, women who reported both douching and genital talc powder use have increased risks of uterine leiomyoma (fibroids), ovarian cancer, and pelvic inflammatory disease than those who only reported using one product, suggesting that cumulative product use may be critical to understanding health risks (39, 41). Despite the evidence of adverse health effects, these products remain poorly regulated with fragmented government oversight. In the USA, the US Food and Drug Administration (FDA) regulates tampons, menstrual pads, menstrual cups, and douching bag apparatuses and nozzles as medical devices whereas douching solutions, sprays, wipes, and powders are regulated as cosmetics (3).

Motivations underlying product choice and behavior are complex and driven by both proximate factors such as peers and family recommendations as well as more distal factors such as intersectional discrimination (e.g., combined discrimination from structural racism, sexism, and classism) (42, 43). In our prior scholarship, we argued that the greater uses of douches among Black women compared to White women may be a consequence of odor discrimination and contribute to the environmental injustice of beauty (43). Historically, perceived mal-odor of African American women by white enslavers has been linked with assertions of sexual immorality and to justify their oppression (44). Later, negative olfactory stereotypes and odor discrimination continued as Black women who failed to adhere to the middleclass archetype of a controlled and disciplined body were denied access to educational and occupational opportunities (1). As a result, Black women were more aggressively marketed products like douches with messaging that encouraged self-consciousness of potential vaginal and vulvar odors and implied healthfulness of product use, despite clinical guidance against douching (44, 45). This practice became embedded within families as a cultural norm, and now persist outside of marketing efforts (44). NHANES data from 2001 to 2004 suggest that more Black women use douches and other intimate care products than white or Mexican American women (36), and the practice of douching is more common among those with less education across all racial/ethnic groups (46). However, current demographic variations in product use are poorly understood given the expansion of the market, and the growth in public awareness about toxic chemicals in personal care products.

Given the socio-historical context, the unique route of chemical exposure, and the lack of regulatory oversight, the objective of our study is to evaluate racial/ethnic and educational differences in use of menstrual and intimate care products among menstruating individuals from two US-based cohort studies. While we examine a range of products, our particular emphasis is on scent-altering products that are marketed for odor control, perceived “freshness”, and cleanliness. Our secondary objective is to examine demographic differences in motivations for product use. We also present descriptive data for non-binary and transgender populations.

## Methods

### Study population

Our analysis combines data from two separate studies of adults aged 18–54 years who reported menstruating in the past year. The Taking Stock Study (TSS) is a community-based participatory research initiative between Occidental College, Black Women for Wellness, local *promotores de salud* (community health workers), Silent Spring Institute, and Columbia University Mailman School of Public Health that examines racial/ethnic differences in consumer product use with a focus on Black women and Latinas using community-generated research questions and collaborative methods of inquiry. We disseminated the TSS survey online to adult (≥18 years) women living in California of all races and ethnicities via online outreach, social media, a Qualtrics panel, and community networks. A detailed description of survey development and dissemination has been described elsewhere (47). The survey was available through Qualtrics between January 2019 and March 2020 in both English and Spanish and completed by 630 participants. Of the 630 participants, we excluded: 15 respondents who did not provide information about menstruating in the past year, and 81 respondents who reported not menstruating in the past year. Thus, data from 534 TSS survey participants are used in the current study. Protocols, including the survey, were reviewed, and approved by Occidental College's Institutional Review Board.

The second study, Fibroids Observational Research on Genes, and the Environment (FORGE), seeks to understand environmental, molecular, and social-structural determinants of gynecologic health conditions, with a specific emphasis on fibroids. In the FORGE study, we recruited and consented individuals who were seeking medical evaluation with the Minimal Invasive Gynecologic Surgery division of the Medical Faculty Affiliates in Washington D.C between 2018 and 2021. We recruited three different groups: (1) individuals who intended to undergo hysterectomy for treatment of non-cancerous, gynecologic conditions (e.g., fibroids, endometriosis); (2) individuals who intended to undergo hysterectomy for gender dysphoria; and (3) individuals newly diagnosed with fibroids. All eligible participants were nonpregnant, premenopausal, and ≥18 years of age. Of the 157 participants enrolled in FORGE, we excluded: 12 participants who did not provide information about menstruating in the past year, 17 participants who reported not menstruating in the past year, and 1 participant who was over 54 years of age. Thus, data from 127 FORGE participants were used in the current study. FORGE study protocols and survey instruments were approved by The George Washington University Institutional Review Board.

### Menstrual and intimate care product use

Both studies used a similar survey design and structure to capture information about menstrual and intimate care product use. Both studies asked participants about their use of three menstrual products (tampons, sanitary napkins/pads, and menstrual cups) and four intimate care products (douches, feminine sprays, feminine powders, and feminine wipes). If the participant reported using a product, they were then asked how frequently they used the product in the past year (less than once a month; 1–3 times a month; during menstrual cycle; 1–5 times a week; 6 or more times per week; and more than once per day). If participants reported using tampons or sanitary pads, they were asked whether their products were scented or unscented. Frequency of menstrual cup use was asked in FORGE but not TSS.

We asked participants about factors that influence their product selection. Questions about participants’ product selection influences were asked differently in the two studies. In FORGE, we asked participants about what influences their product use in a single question. In TSS, we asked two questions: (1) what characteristics are important when choosing a product and (2) where do you go to learn more about products.

### Data harmonization

In addition to questions about product use, we asked several questions about the participants’ demographics. All participants self-identified their race/ethnicity and gender, with an option not to disclose. Data from TSS and FORGE were harmonized to create a unified dataset for the analysis. In general, FORGE data were adjusted to match the survey structure and available responses from TSS survey prior to merging the data. For example, FORGE participants who identified as “Woman” were reported here as “Female” to be in parallel with the identities reported by TSS participants (and because all FORGE participants were assigned female at birth). Age was asked differently in the two studies; TSS participants selected an age category (i.e., 18–24 years, 25–34 years, 35–44 years, and 45–54 years) whereas age was calculated for FORGE participants based on their date of birth abstracted from medical records. As a result, age categories are used in the current analysis. We categorized self-identified race/ethnicity as non-Hispanic Black/African American (“Black”), Hispanic/Latinx (“Latinx”), non-Hispanic White/Caucasian (“White”), or some other identity. Latinx includes any participant who identified as Latinx even if they also reported another racial/ethnic identity. Some other identity captures those who identify with racial/ethnic groups other than Black, White, or Latinx (e.g., Asian, American Indian) as well as multiracial participants. We also asked participants about their level of formal education. We categorized self-reported formal education attainment into three categories: ≤high school graduate or GED credential (abbreviated as ≤high school diploma), some college, technical school or associate degree (abbreviated as some college), or ≥bachelor's degree. Lastly, we categorized self-identified sex/gender into three categories: female, transgender, and non-binary.

### Data analysis

We summarized product use (yes vs. no) by participant demographics and evaluated differences in product use by each demographic variable using the Fisher's exact test. We used frequency of use data to determine whether participants used products largely during menstruation or as a more regular practice. To summarize and compare the frequency of product use, we collapsed the frequency data into three categories: occasionally (e.g., less than once a month or 1–3 times a month), during menstrual cycle, or regularly (e.g., 1–5 times a week, 6 or more times per week, or more than once per day). We assessed concordance in product use for each pair of products using the phi coefficient. We *a priori* identified race/ethnicity, education, age, sex/gender, and study (FORGE vs. TSS) as important covariates. To evaluate the association between product use and each covariate, we used relative risk (log-link) binomial regression models. We first modeled each covariate of interest and product use (outcome variable) separately. We then combined education, race/ethnicity, age, and study within a multivariate model. Due to the small sample size of non-binary and transgender participants, we only examined differences in product use by sex/gender using descriptive statistics. We reported relative risks for each covariate from the mutually adjusted models. We used the fully adjusted log-binomial models to predict probabilities of use of each product for each category of race/ethnicity and education.

We used latent class analysis (LCA) to identify groups of participants with similar product use patterns, using an approach similar to Wang et al. (48) We used multiple criteria to assess the fit of the LCA model, including Bayesian Information Criterion and minimum class size (at least 10%). We selected the most parsimonious model, which was a model with two latent classes. We then categorized the two classes based on the probability of use of different products. Next, we summarized demographic characteristics for each latent class and assessed differences using Fisher's exact test. We further examined determinants of latent class assignment membership by regressing race/ethnicity, education, study, and age against the predicted class assignment in a multivariate model. Lastly, we examined differences in influences on product selection by LCA class assignment using Fisher's exact test.

We conducted sensitivity analyses to evaluate the relationship between educational attainment and product use in the subset because many population health and census studies only examine educational attainment as a risk factor among those who have completed their education and most US adults have completed their formal education by 25 years of age (49, 50). To explore the relationship between formal education and product use, we reran all mutually adjusted log-binomial models for individual product use among those ages 25–54. Additionally in this subset, we re-ran the LCA model and re-examined differences in probability of being in a certain LCA class by all demographic variables. In the case of scented tampon and scented pad use, 13 and 31 participants, respectively, were unsure about whether the products they used were scented. In the main analysis, we included unsure respondents with the “no” respondents. As a sensitivity analysis, we removed those who were unsure from the analysis. Since the results from the two analyses were similar, we only show results from models where the unsure participants are grouped with the “no” participants for scented menstrual product use.

## Results

### Descriptive characteristics of study population and product use

Our study population consisted of 661 participants aged 18–54 years (Table 2). Approximately 80% of the population were from TSS (*N* = 534) and 20% was from FORGE (*N* = 127). TSS participants were younger than FORGE participants, with 59.3% of TSS participants aged 18–34 years compared 12.5% of FORGE participants. Most respondents identified as female (*n* = 640); however, the population also included a small sample who identified as transgender (*n* = 9 or 1.4%) or non-binary (*n* = 12 or 1.8%). The most common racial/ethnic group was Black (33.4%) and followed by Latinx (30.9% of overall). Racial/ethnic composition varied by study with Black participants being the largest subpopulation in FORGE and Latinx participants being the largest subpopulation in TSS. Across both studies, 3.5% did not complete high school, 16.2% had a high school diploma or equivalent, 34.2% reported some college or an associate/technical degree, and 45.0% reported ≥bachelor's degree. The FORGE population had more formal education than the TSS population.

**Table 2:** Demographic characteristics of study participants by study (number and %).

	FORGE (*N* = 127)	TSS (*N* = 534)	Overall (*N* = 661)
Age (years)			
18–24	4 (3.1%)	185 (34.6%)	189 (28.6%)
25–34	12 (9.4%)	132 (24.7%)	144 (21.8%)
35–44	61 (48.0%)	154 (28.8%)	215 (32.5%)
45–54	50 (39.4%)	63 (11.8%)	113 (17.1%)
Race			
Black	88 (69.3%)	133 (24.9%)	221 (33.4%)
Latinx	6 (4.7%)	198 (37.1%)	204 (30.9%)
White	27 (21.3%)	93 (17.4%)	120 (18.2%)
Some other identity	6 (4.7%)	101 (18.9%)	107 (16.2%)
Missing	0 (0.0%)	9 (1.7%)	9 (1.4%)
Education			
Less than high school diploma	5 (3.9%)	18 (3.4%)	23 (3.5%)
High school diploma/GED	16 (12.6%)	91 (17.0%)	107 (16.2%)
Some college	31 (24.4%)	166 (31.1%)	197 (29.8%)
Technical school/associate degree	4 (3.1%)	25 (4.7%)	29 (4.4%)
Bachelor’s degree	32 (25.2%)	129 (24.2%)	161 (24.4%)
Graduate degree	35 (27.6%)	101 (18.9%)	136 (20.6%)
Missing	4 (3.1%)	4 (0.7%)	8 (1.2%)
Sex/gender			
Female	116 (91.3%)	524 (98.1%)	640 (96.8%)
Transgender	7 (5.5%)	2 (0.4%)	9 (1.4%)
Non-binary	4 (3.1%)	8 (1.5%)	12 (1.8%)

**Figure 1:**
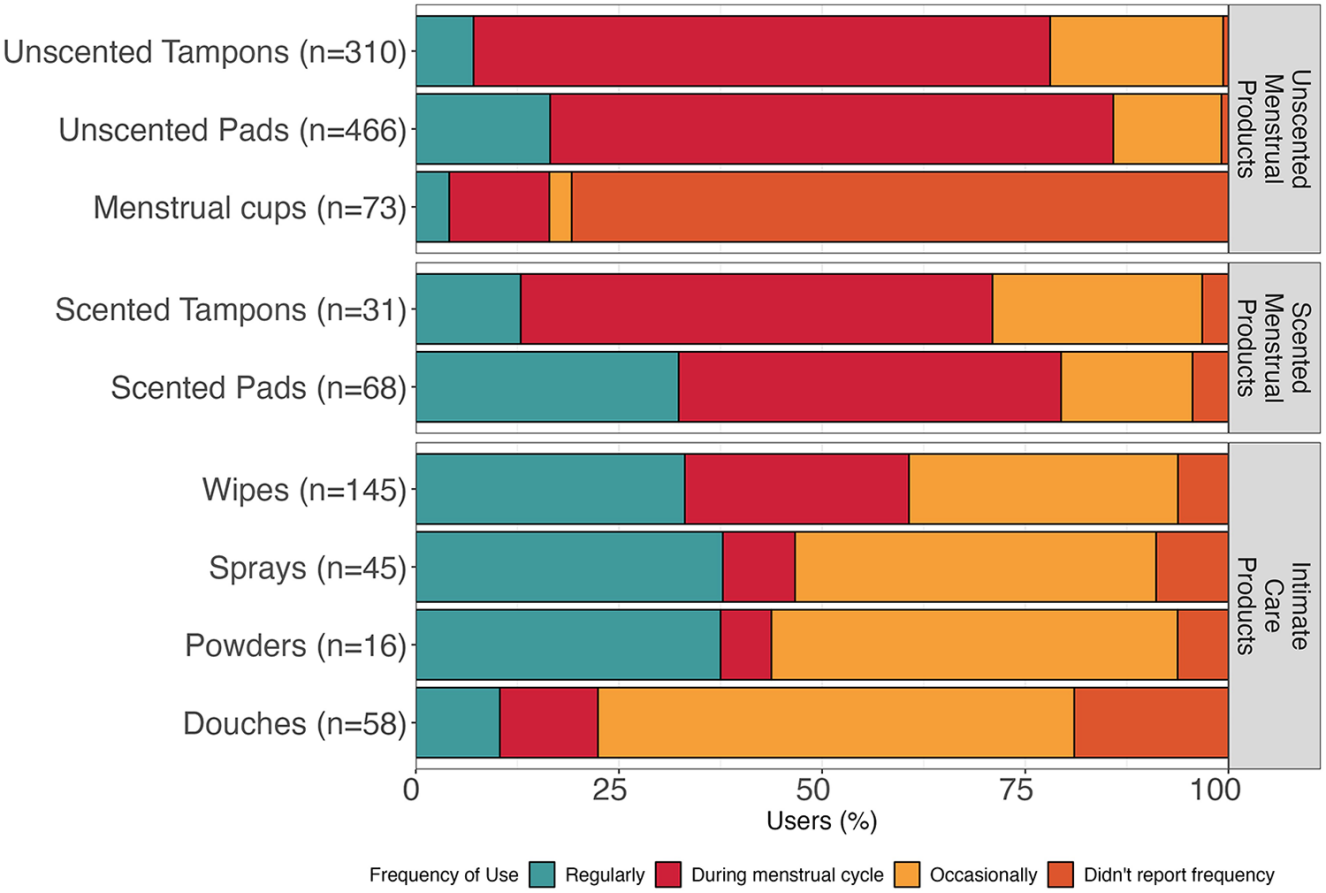
Reported frequency of use of menstrual and intimate care products for all participants. Occasionally indicates less than three times a month and Regularly at least once per week.

Unscented menstrual products were the most used products; 70.0% of participants reported using unscented pads and 47.0% reported using unscented tampons (Table 3). Menstrual cups were used by 11.0% of the population. Scented pads were used more commonly than scented tampons (10.0% vs. 4.7%). Among intimate care products, wipes were the most common (22.0% of participants), followed by douches (8.8%), sprays (6.8%), and powders (2.4%). Figure 1 shows frequency of product use among users. While menstrual products were most used during menstruation, some participants reported more frequent use. For example, one-third of scented pad users reported using these products regularly (i.e., at least once per week). Relatedly, 7.1% of unscented tampon users and 12.9% of scented tampon users reported using these products regularly. In general, intimate care products were used more regularly than menstrual products. For example, wipes, sprays, and powders were used regularly by 30%–40% of users. In contrast, douches were more likely to be used occasionally (i.e., less than three times a month) (Figure 1). Use of scented menstrual products (tampons and pads) was positively correlated with use of intimate care products (douches, sprays, wipes and powders), whereas use of unscented menstrual products was negatively correlated with intimate care product use (Supplementary Figure S1).

**Table 3:** Product use (number and %) by demographic characteristics.

	Unscented pads	Unscented tampons	Menstrual cups	Scented pads	Scented tampons	Wipes	Douches	Sprays	Powders
Total (*N* = 661)	466 (70.0%)	310 (47.0%)	73 (11%)	68 (10%)	31 (4.7%)	145 (22.0%)	58 (8.8%)	45 (6.8%)	16 (2.4%)
Age (years)									
18–24 (*N* = 189)	125 (61.1%)	99 (52.4%)	**28** (14.8%)*	13 (6.9%)	7 (3.7%)	**21** (11.1%)*	**5** (2.6%)*	**5** (2.6%)*	2 (1.1%)
25–34 (*N* = 144)	109 (75.7%)	69 (47.9%)	**24** (16.7%)*	10 (6.9%)	4 (2.8%)	**34** (23.6%)*	**11** (7.6%)*	**12** (8.3%)*	2 (1.4%)
35–44 (*N* = 215)	150 (69.8%)	94 (43.7%)	**18** (8.4%)*	30 (14.0%)	13 (6.0%)	**58** (27.0%)*	**25** (11.6%)*	**23** (10.7%)*	9 (4.2%)
45–54 (*N* = 113)	82 (72.6%)	48 (42.5%)	**3** (2.7%)*	15 (13.3%)	7 (6.2%)	**32** (28.3%)*	**17** (15.0%)*	**5** (4.4%)*	3 (2.7%)
Race/ethnicity									
Black (*N* = 221)	150 (67.9%)	**83** (37.6%)*	**13** (5.9%)*	22 (10.0%)	13 (5.9%)	**72** (32.6%)*	**34** (15.4%)*	22 (10.0%)	8 (3.6%)
Latinx (*N* = 204)	154 (75.5%)	**86** (42.2%)*	**19** (9.3%)*	22 (10.8%)	9 (4.4%)	**42** (20.6%)*	**17** (8.3%)*	15 (7.4%)	2 (1.0%)
White (*N* = 120)	76 (63.3%)	**84** (70.0%)*	**27** (22.5%)*	10 (8.3%)	4 (3.3%)	**16** (13.3%)*	**3** (2.5%)*	4 (3.3%)	5 (4.2%)
Some other identity (*N* = 107)	80 (74.8%)	**52** (48.6%)*	**11** (10.3%)*	13 (12.1%)	5 (4.7%)	**15** (14.0%)*	**4** (3.7%)*	4 (3.7%)	1 (0.9%)
Education									
≤High school diploma (*N* = 130)	**80** (61.5%)*	**42** (32.3%)*	**5** (3.8%)*	**23** (17.7)*	10 (7.7%)	34 (26.2%)	**23** (17.7%)*	**17** (13.1%)*	4 (3.1%)
Some college, technical school, or associate degree (*N* = 226)	**156** (69.0%)*	**104** (46.0%)*	**23** (10.2%)*	**25** (11.1%)*	11 (4.9%)	50 (22.1%)	**18** (8.0%)*	**17** (7.5%)*	9 (4.0%)
≥Bachelor’s degree (*N* = 297)	**226** (76.1%)*	**164** (55.2%)*	**45** (15.2%)*	**19** (6.4%)*	9 (3.0%)	58 (19.5%)	**14** (4.7%)*	**9** (3.0%)*	3 (1.0%)
Sex/gender									
Female (*N* = 640)	455 (71.1%)	297 (46.4%)	**66** (10.3%)*	67 (10.5%)	31 (4.8%)	141 (22.0%)	55 (8.6%)	45 (7.0%)	16 (2.5%)
Transgender (*N* = 9)	5 (55.6%)	5 (55.6%)	**0** (0.0%)*	1 (11.1%)	0 (0.0%)	2 (22.2%)	22 (22.2%)	0 (0.0%)	0 (0.0%)
Non-binary (*N* = 12)	6 (50%)	8 (66.7%)	**7** (58.3%)*	0 (0.0%)	0 (0.0%)	2 (16.7%)	1 (8.3%)	0 (0.0%)	0 (0.0%)
Study									
TSS (*N* = 534)	373 (69.9%)	248 (46.4%)	59 (11.0%)	52 (9.7%)	23 (4.3%)	**107** (20.0%)*	**38** (7.1%)*	35 (6.6%)	13 (2.4%)
FORGE (*N* = 127)	93 (73.2%)	62 (48.8%)	14 (11.0%)	16 (12.6%)	8 (6.3%)	**38** (29.9%)*	**20** (15.7%)*	10 (7.9%)	3 (2.4%)

Differences by demographic variable evaluated using a fisher test, with statistically significant differences (*p* < 0.05) in bold*.

### Associations between sociodemographic variables and product use

Product use varied by age, race/ethnicity, education, and sex/gender, and study in unadjusted, bivariate analyses (Table 3). Menstrual cup use was highest among the 18–24 years age group and significantly declined in the older age groups. Whereas use of wipes, douches, and sprays generally increased with age. Use of four products (unscented tampons, menstrual cups, douches, and wipes) varied by race/ethnicity. Use of unscented tampons and menstrual cups were most common among White participants with 70.0% and 22.5% reporting use, respectively. Whereas use of wipes and douches was highest among Black participants with 32.6% and 15.4% reporting use, respectively. Most products significantly varied by education; unscented menstrual product use was more common among those with ≥bachelor's degree whereas scented menstrual and intimate care product use was generally more common among those with ≤high school diploma. There were also significant differences in menstrual cup use by sex/gender with most non-binary participants (58.3%) reporting use compared to 10.3% among other female participants. None of the non-binary or transgender participants reported using scented tampons, sprays, or powders.

**Figure 2:**
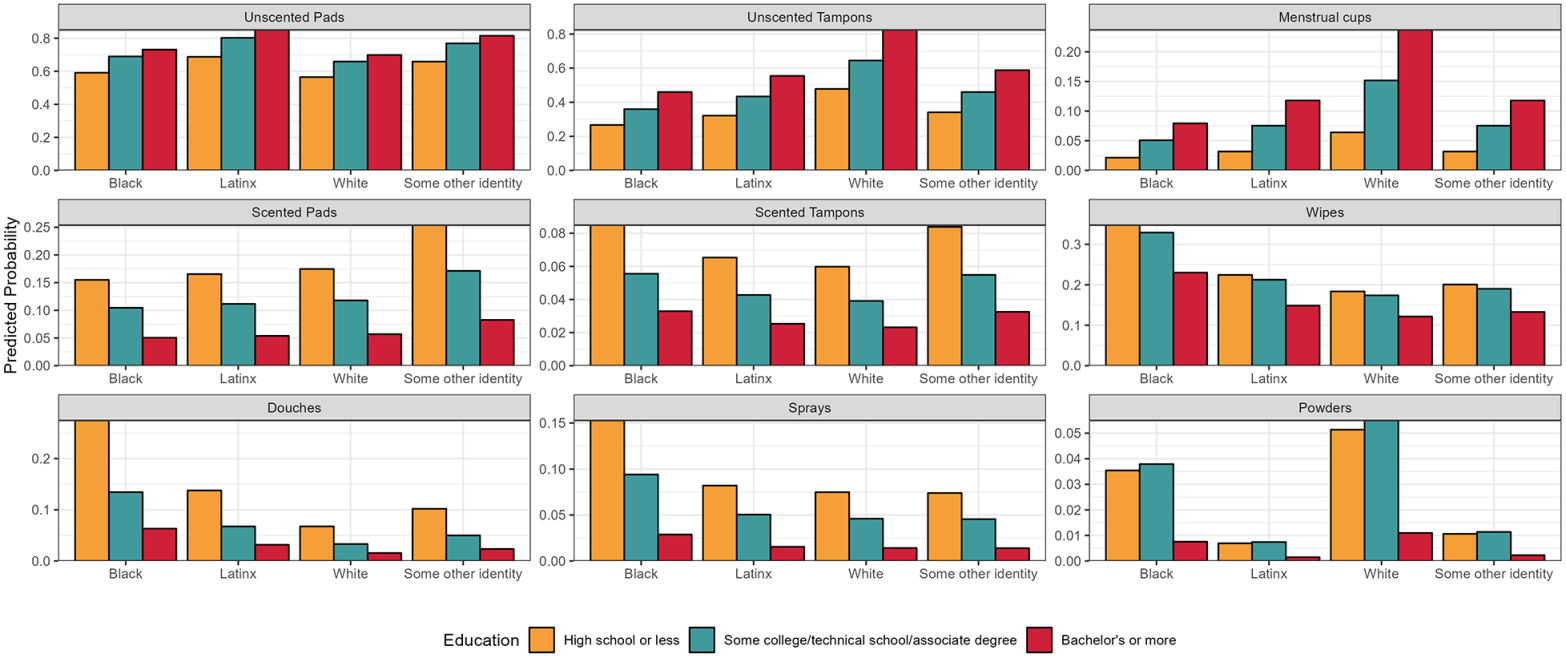
Predicted probability of product usage by educational attainment and by race (*N* = 644).

In mutually adjusted log-binomial models, age, race/ethnicity, and education remained important determinants of product use (Table 4). Compared to 18–24 year age group, there was decreased risk of menstrual cup use among 35–44 year age group (relative risk (RR) = 0.49, 95% confidence intervals (CI): 0.26, 0.93) and 45–54 year age group (RR = 0.16, 95% CI: 0.047, 0.52). However, there was increased risks of use of scented pads, wipes, douches, sprays, and powders among older participants, particularly in the 35–44 year age group compared to 18–24 age group. Compared to Black participants, White participants had a 1.8 (95% CI: 1.5, 2.2) and a 3.0 (95% CI: 1.1, 5.6) relative risk of using unscented tampons and menstrual cups, respectively. Black participants had significantly higher relative risks of using wipes compared to all other racial/ethnic groups. Similarly, Black participants had higher risks of douche use compared to White (RR = 0.25, 95% CI: 0.076, 0.80) and Latinx (RR = 0.5, 95% CI: 0.27, 0.94) participants. Black participants also had higher risk of use of powders compared to Latinx participants. Furthermore, in adjusted models, educational attainment became a significant determinant of each product used. There were increased risks of use of unscented pads, unscented tampons, and menstrual cups among those with ≥bachelor's degree compared to those with those with ≤high school diploma. In contrast, use of scented menstrual and intimate care products showed an inverse relationship with formal education with the greatest risks of use among those with the least formal education. For example, use of scented pads (RR = 3.1, 95% CI: 1.7, 5.6), scented tampons (RR = 2.6, 95% CI: 1.0, 6.4), wipes (RR = 1.5, 95% CI: 1.0, 2.2), douches (RR = 4.3, 95% CI: 2.3, 8.2), sprays (RR = 5.3, 95% CI: 2.4, 12), and powders (RR = 4.7 (1.1, 21) was associated with increased risks among ≤high school diploma compared to ≥bachelor's degrees. In most cases, those with some college had an intermediate risk of use between those with ≤high school diploma and those with ≥bachelor's degree. Figure 2 shows the predictive probability of product use by race/ethnicity and education. Study was not associated with any product use in mutually adjusted models.

**Table 4:** Relative risks (95% CIs) from mutually adjusted log-binomial models.

Term	Unscented pads	Unscented tampons	Menstrual cups	Scented pads	Scented tampons	Wipes	Douches	Sprays	Powders
Age (years)									
18–24 (*N* = 189)	REF	REF	REF	REF	REF	REF	REF	REF	REF
25–34 (*N* = 144)	1.1 (0.95, 1.3)	**0.80** (0.66, 0.98)*	0.96 (0.55, 1.7)	1.4 (0.58, 3.2)	0.95 (0.27, 3.4)	**2.3** (1.4, 4)*	**3** (1.1, 8.7)*	**4.1** (1.4, 12)*	3.2 (0.43, 23)
35–44 (*N* = 215)	1 (0.89, 1.2)	**0.86** (0.72, 1)*	**0.49** (0.26, 0.93)*	**2.5** (1.3, 4.9)*	1.6 (0.6, 4.5)	**2.3** (1.4, 3.8)*	**2.9** (1.1, 7.9)*	**4.1** (1.5, 11)*	**7.8** (1.6, 38)*
25–54 (*N* = 113)	1 (0.87, 1.2)	0.87 (0.71, 1.1)	**0.16** (0.047, 0.52)*	**2.6** (1.2, 5.5)*	1.9 (0.61, 6)	**2.4** (1.4, 4.1)*	**4.4** (1.6, 12)*	1.5 (0.39, 5.8)	5.1 (0.81, 31)
Race									
Black (*N* = 221)	REF	REF	REF	REF	REF	REF	REF	REF	REF
Latinx (*N* = 204)	**1.2** (1, 1.3)*	1.3 (0.98, 1.6)	1.5 (0.75, 3)	1.1 (0.56, 2)	0.77 (0.3, 2)	**0.65** (0.45, 0.93)*	**0.5** (0.27, 0.94)*	0.54 (0.26, 1.1)	**0.2** (0.039, 0.98)*
White (*N* = 120)	0.96 (0.81, 1.1)	**1.9** (1.5, 2.3)*	**3** (1.6, 5.6)*	1.1 (0.54, 2.4)	0.7 (0.22, 2.2)	**0.53** (0.32, 0.87)*	**0.25** (0.076, 0.8)*	0.49 (0.17, 1.4)	1.5 (0.46, 4.5)
Some other identity (*N* = 107)	1.1 (0.96, 1.3)	**1.3** (1, 1.8)*	1.5 (0.68, 3.3)	1.6 (0.8, 3.4)	0.99 (0.32, 3)	**0.58** (0.34, 0.97)*	0.37 (0.13, 1.1)	0.48 (0.17, 1.4)	0.3 (0.036, 2.5)
Education									
≤High school diploma (*N* = 130)	**0.81** (0.69, 0.94)*	**0.53** (0.39, 0.7)*	**0.27** (0.11, 0.67)*	**3.1** (1.7, 5.6)*	**2.6** (1, 6.4)*	**1.5** (1, 2.2)*	**4.3** (2.3, 8.2)*	**5.3** (2.4, 12)*	**4.7** (1.1, 21)*
Some college, technical school, or associate degree (*N* = 226)	0.94 (0.84, 1.1)	**0.79** (0.67, 0.94)*	0.64 (0.38, 1.1)	**2.1** (1.1, 3.7)*	1.7 (0.69, 4.1)	**1.4** (1, 2)*	**2.1** (1.1, 4.2)*	**3.3** (1.5, 7.2)*	**5** (1.4, 18)*
≥Bachelor’s degree (*N* = 297)	REF	REF	REF	REF	REF	REF	REF	REF	REF
Study									
TSS (*N* = 534)	REF	REF	REF	REF	REF	REF	REF	REF	REF
FORGE (*N* = 127)	1.1 (0.96, 1.3)	1.2 (1.0, 1.4)	1.5 (0.82, 2.6)	1.2 (0.62, 2.2)	0.94 (0.35, 2.5)	0.96 (0.67, 1.4)	0.96 (0.51, 1.8)	0.73 (0.33, 1.6)	0.41 (0.11, 1.5)

CIs, Confidence Interal; REF, Referent Group.

Statistically significant differences (*p* < 0.05) from the referent group are indicated by an asterisk and bold (*N* = 644).

### Unscented and scent-altering product use

**Figure 3:**
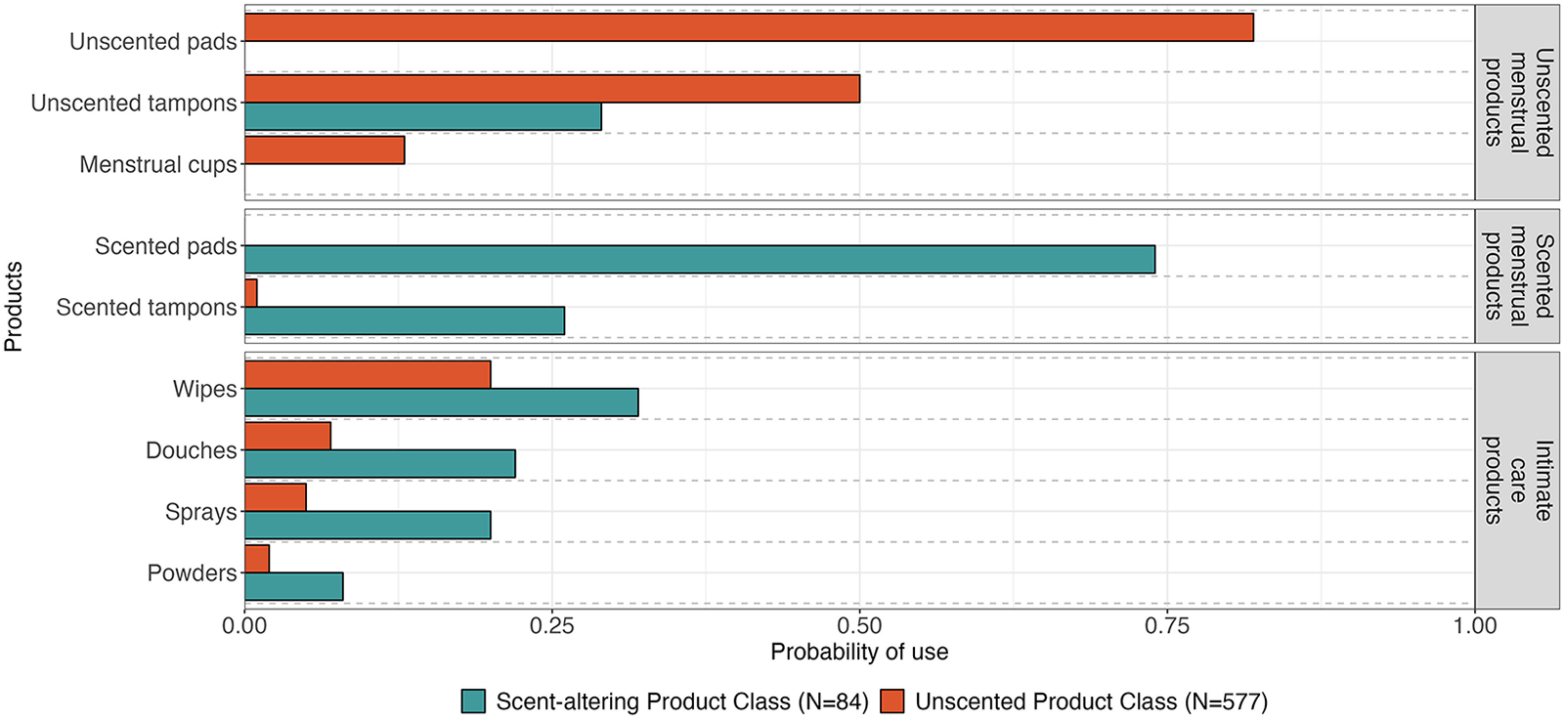
Product use probability by latent class.

**Figure 4:**
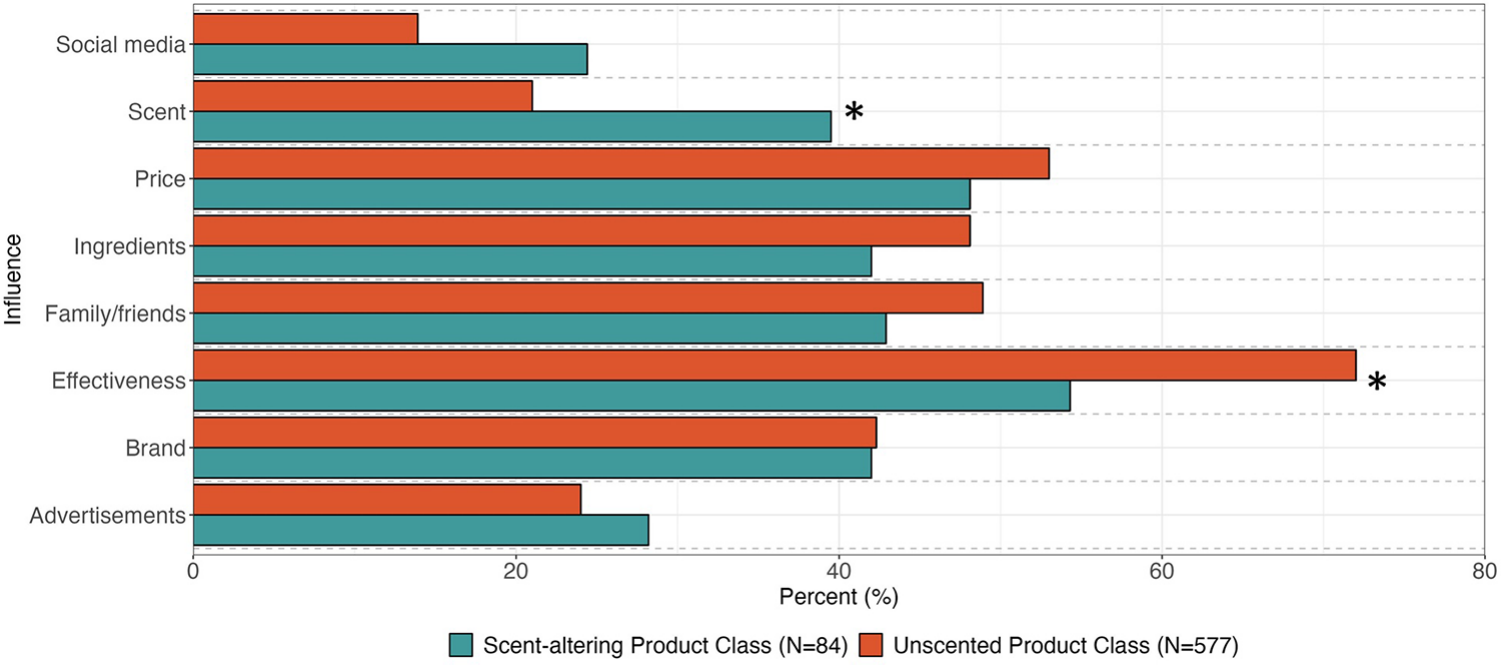
Influences impacting product selection stratified by latent class assignment. Significant differences (*p* < 0.05) indicated with asterisk.

**Figure 5:**
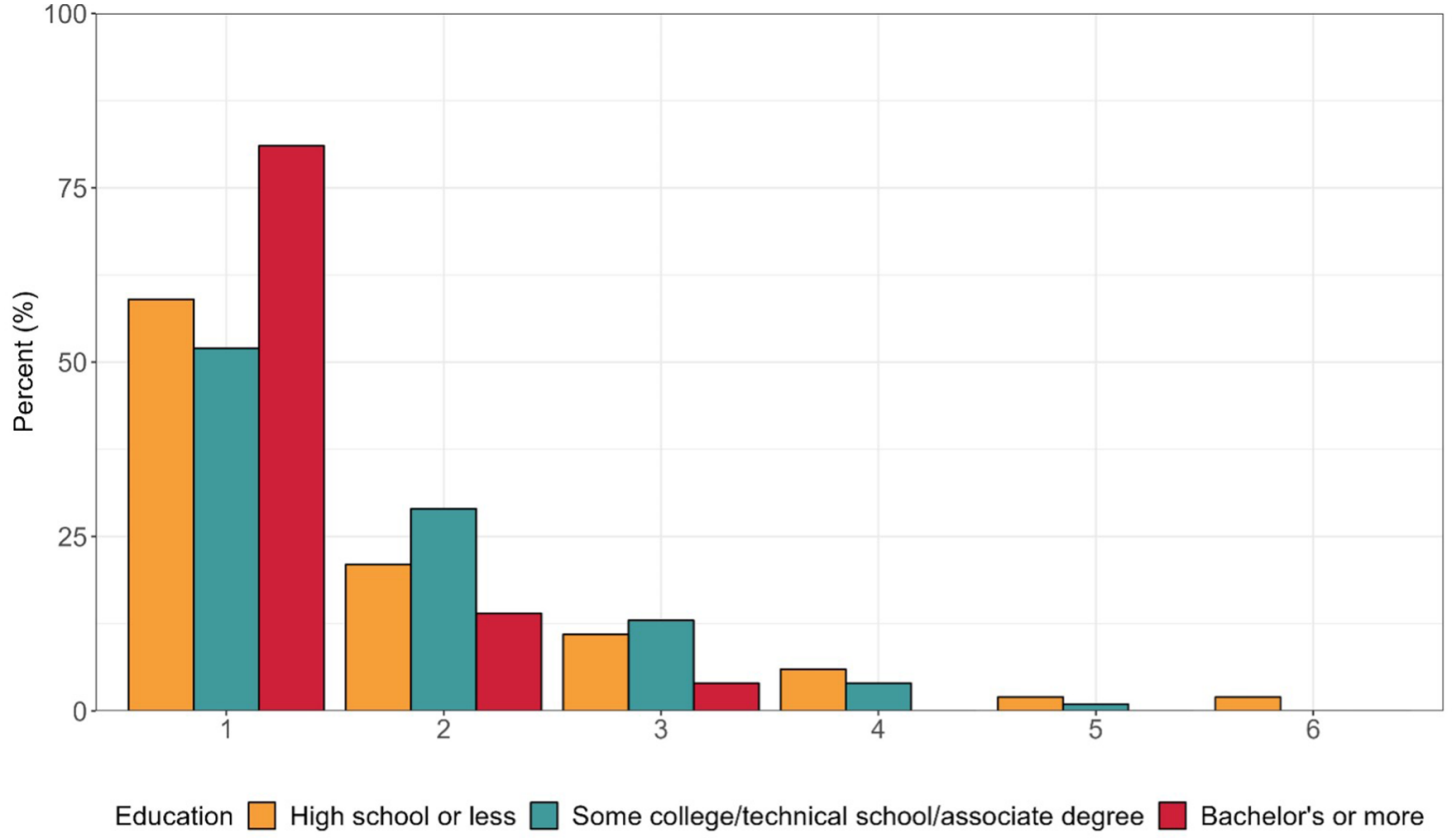
Number of scent-altering products used among those who reported using at least one scent-altering product stratified by education: high school or less (*n* = 63), some college/technical school/associate degree (*n* = 75) and bachelor’s or more (*n* = 91).

We next sought to understand whether product use could distinguish groups of participants (i.e., do respondents cluster based on their reported product use) and if those groups varied by demographic characteristics. The LCA identified two distinct classes or groups of product users. The first group (*n* = 84) was more likely to use scent-altering products, including scented menstrual care products as well as the four intimate care products. The second group (*n* = 577) was more likely to use unscented tampons, unscented pads, and menstrual cups (Figure 3). There were clear differences in age and formal education between the two groups (Table 5). Most participants in the scent-altering product use class were between the ages of 35–54 (64.3%) and had less than a bachelor's degree (71.0%). Age and formal education remained significant determinants of membership in scent-altering product class in log-binomial regression models after adjustment for race/ethnicity and study. For example, compared to those with ≥bachelor's degree, those with ≤high school diploma and those with some college had relative risks of 3.2 (1.8, 5.4) and 2.1 (1.2, 3.6), respectively, of having membership in the scent-altering product class. None of the other demographic factors varied by class assignment. While sex/gender did not statistically vary between the two groups, all the respondents who identified as non-binary fell within the group less likely to use scent-altering products. Those who belonged to the scent-altering LCA class were more likely to report choosing products based on their scent compared to the other class (Figure 4). Whereas those in the unscented product class were more likely to report choosing products based on effectiveness. Figure 5 shows the distribution of number of scent-altering product use among those who reported using at least one product by three education categories. Those with ≤high school diploma reported using more scent-altering products than those with more formal education. Among those with ≤high school diploma, 9.5% reported using four or more scent-altering products.

**Table 5:** Demographic differences by latent class.

	Scent-altering product class		
Characteristic	Yes (*N* = 84)	No (*N* = 577)	Adjusted RR (95% CI)
Age (years)			
18–24 (*N* = 189)	17 (20.2%)	172 (29.8%)	REF
25–34 (*N* = 144)	13 (15.5%)	131 (22.7%)	1.4 (0.67, 2.9)
35–44 (*N* = 215)	36 (42.9%)	179 (31.0%)	**2.3** (1.2, 4.1)*
45–54 (*N* = 113)	18 (21.4%)	95 (16.5%)	**2.4** (1.2, 4.7)*
Race/ethnicity^a^			
Black (*N* = 221)	31 (37.3%)	190 (33.4%)	
Latinx (*N* = 204)	26 (31.3%)	178 (31.3%)	0.79 (0.45, 1.4)
White (*N* = 120)	13 (15.7%)	107 (18.8%)	0.98 (0.53, 1.8)
Some other identity (*N* = 107)	13 (15.7%)	94 (16.5%)	1 (0.52, 1.9)
Education^b^			
≤High school diploma (*N* = 130)	28 (34.1%)	102 (17.9%)	**3.2** (1.8, 5.4)*
Some college, technical school, or associate degree (*N* = 226)	31 (37.8%)	195 (34.2%)	**2.1** (1.2, 3.6)*
≥Bachelor’s degree (*N* = 297)	23 (28.0%)	274 (48.0%)	REF
Sex/gender			
Female (*N* = 640)	83 (98.8%)	557 (96.5%)	NA
Transgender (*N* = 9)	1 (1.2%)	8 (1.4%)	NA
Non-binary (*N* = 12)	0 (0.0%)	12 (2.1%)	NA
Study			
TSS (*N* = 534)	66 (78.6%)	468 (81.1%)	REF
FORGE (*N* = 127)	18 (21.4%)	109 (18.9%)	0.82 (0.46, 1.4)

RR, Relative Risk; CI, Confidence Intervals; REF, Referent Group.

Adjusted RRs (95% CI) are for relative risk of having membership in the scent-altering product class. Significant differences (*p* < 0.05) in bold, (*N* = 661).

^a^ 9 participants did not report race/ethnicity.

^b^ 8 participants did not report education.

### Sensitivity analysis

As a sensitivity analysis, we further examined the association between education and product use among those 25 years and older, when most US adults have typically completed their formal education. We first examined the relative risks of each individual product use from mutually adjusted log-binomial models. Associations between education and individual product use were generally consistent with those from the main analysis except for scented tampons, which was not associated with education in the main analysis. In the sensitivity analysis, there was a significant association between scented tampon use and education. Compared to those with ≥bachelor's degree, there is an increased risk of use of scented tampons among those with ≤high school diploma (RR = 4.6, 95% CI: 1.6, 13) and some college (RR = 3.0, 95% CI: 1.1, 8.3) (Supplemental Table S1). We also reran the LCA and re-examined demographic determinants of LCA class assignment in the older subset. The adjusted association between educational attainment and scent-altering product class assignment was more pronounced in the sensitivity analysis. Compared to those with ≥bachelor's degrees, those with ≤high school diploma had a relative risk of 4.4 (95% CI: 2.4, 7.9), compared to a relative risk of 3.2 (95% CI: 1.8, 5.4) in the main analysis. Age was not a significant determinant of scent-altering product class membership in the restricted analysis.

## Discussion

In this pooled analysis of two US-based study populations, we found a consistent relationship between level of formal education and use of menstrual and intimate care products: those with the less formal education were more likely to use multiple scent-altering products and those with more formal education were more likely to use unscented tampons and menstrual cups. We also found racial/ethnic differences in product use; compared to Black participants, White participants were more likely to use unscented tampons and menstrual cups and less likely to use douches and wipes. We observed important differences by age; those who were ages 18–24 were more likely to use menstrual cups and less likely to use intimate care products. Lastly, we present some of the first data on product use among gender minorities with descriptive statistics on transgender and nonbinary respondent product usage. Among the small sample of non-binary participants, there was a high prevalence of use of menstrual cups and no reported use of scented menstrual products, powders, or sprays. Collectively, our findings suggest importance differences in menstrual and intimate care product use by measures of social identity (e.g., race/ethnic, socioeconomic, and gender identities) further underscoring the significance of the menstrual and intimate care industry to the environmental injustice of beauty.

### Consistency with prior literature

There is limited prior research upon which we can contextualize our research findings. To our knowledge, no prior studies have characterized demographic differences in usage of menstrual cups, scented tampons, or scented pads aside from Dodson et al. who examined racial/ethnic differences in personal care products use among TSS participants ages 18–34. (47) Among the products included in this study, sociodemographic patterns in douching are the most well characterized. Prior literature suggests that douching is most common in Black women, specifically among those who are lower socioeconomic status, as reflected by their education or income (46, 51, 52). Our findings are consistent with those from previous studies. Moreover, the douching prevalence for Black participants in our study (data collected from 2018 to 2021) was similar to estimates from Branch et al. (NHANES 2001–2004) (34% vs. 37%) despite efforts by the clinical community to discourage this practice (36). Branch et al. also reported that Black women used significantly more wipes, sprays, and powders than White or Mexican American women. We found similar patterns although some of the differences between racial/ethnic groups in our study did not reach statistical significance.

We found significant differences in racial/ethnic patterns of use of unscented tampons and menstrual cups, both of which were more likely to be used by White, more highly educated, and younger participants. In contrast, there were no racial/ethnic differences in use of scented pads or tampons. Previous literature supports our findings on unscented tampons and menstrual cups: one study demonstrated that White women are more likely to report using tampons as adolescents compared to Black and Latina peers, which was credited to differences in household and cultural norms (53). Similarly, menstrual cup usage has been reported to be higher amongst younger populations with greater educational attainment among participants ages 18–55 in Spain (54). We also found menstrual cup use was highly prevalent among our small sample of non-binary participants, despite this product generally having lower reported usage rates across literature (55). While our study cannot elucidate upstream drivers of product use within this group, an ethnographic study of 19 trans and non-binary participants found that respondents chose menstrual products with respect to their gender identities and body politics, with many opting for products that minimized gender dysphoria during menstruation (56).

### Educational variation in scent-altering product use

We found that use of all six scent-altering products were separately associated with less education in the fully adjusted relative risk models, and that combined use of these products was also more common in those with less education. Consistent with the product use findings, those who belonged to the “scent-altering” latent class were also more likely to report scent as an influential factor in product choice and selection. To our knowledge, ours is the most in-depth examination of educational variation of menstrual and intimate care product use.

The cultural marketing and use of menstrual products in the US shifted menstruation from a natural function and aspect of fertility into a hygiene crisis that needed to be managed by scientists, the medical community, and menstrual product manufacturers (1). The vagina has been historically described by advertisers as having a negative odor and in need of deodorizing, disinfecting, and cleaning, promoting sales of douching products to women for “freshness” and marital harmony. From the earliest commercial menstrual products in the early twentieth century, ads reinforced two notions—that menstruation was a hygiene issue and an odor issue. Tampon and pad manufacturers added perfume and scents to their products to “protect against odor.” (57) The rise of synthetic fragrance manufacturing intersects with the post war rise of the petrochemical industry, and the marketing of a range of products to US consumers, including single use products, plastics, and other throw away items (58, 59).

Our study cannot directly disentangle how formal education interacts with the cultural history around scent and odor in the U.S. It is possible that attainment of formal education could be a proxy for differences in cultural norms and social taboos by socioeconomic status. While we did not measure socioeconomic status during childhood, extensive prior data demonstrates an association between socioeconomic status during childhood and adulthood (60). Social taboos surrounding menstruation and odor can come from media, religion, and cultural norms, and can largely influence what types of products people use (61). Most women report that guidance on menstrual hygiene is shaped during their adolescent years and are strongly influenced by their mother and other family members (62). As such, the relative importance of social taboos around body odor could vary across the socioeconomic spectrum. Alternatively, our results suggest that college and post-graduate education can expose menstruating populations to additional information about reproductive and menstrual health beyond what they learned in high school, including broader exposure to menstrual activism and other social movements that have sought to reframe the symbolism of menstruation and messages in menstrual product marketing (63). Social movements surrounding the normalization of menstruation have inspired art, humor, legislation, and campus activism. For example, in recent years activists have successfully drawn critical attention to 35 states which impose a sales tax on menstrual hygiene products, while products such as those for erectile dysfunction are tax-free. This “tampon tax” has become emblematic of gender inequality, as it imposes a burden on top of the purchase of biologically necessary products that menstruating individuals require to attend work, school, and participate in public life (64). Furthermore, for many, college might be the first time living with non-family members. The most recent data available from the National Center for Education Statistics suggests that the majority of bachelor's degree seeking students live outside of their family home, either on campus (29.1%) or off campus with roommates (42.6%) (65). Comparatively, 40.0% of students enrolled in 2-year associate degree programs report residing with parents. For many, college might be the first exposure to broader menstrual equity conversations and residing with non-family members, which can potentially create opportunities for dialogue about alternative menstrual management products and dispel myths about odor and hygiene (66, 67). Future research should further investigate the mechanisms underlying our observed association between education and scent-altering product use.

### Exposure and health implications

The potential health implications of our findings warrant further consideration. Few toxicologic or epidemiologic studies have considered the adverse health risks of menstrual and intimate care product use in relation to racial/ethnicity or education. Available risk assessments are limited since most have only estimated health risks from one class of chemicals in one product (e.g., cancer risks of VOCs in sprays) (16, 35). Our study importantly highlighted that some menstruating individuals are using multiple (up to six) scent-altering products in and around vaginal and vulvar tissues. Many of these products contain fragrance chemicals. Use of fragranced products on vulvar tissue warrants unique consideration since vulvar tissue differs from cutaneous epithelia in structure, morphology, and biophysical characteristics. For example, the skin of the labia majora exhibits unique hydration, occlusion, and frictional properties, which may increase susceptibility to irritants and contact sensitizers. Furthermore, the nonkeratinized vulvar vestibule is likely to be more permeable than keratinized regions found in other parts of the body. These differences heighten vulvar susceptibility to topical agents including chemicals in intimate care products, which have been reported sources of allergic contact dermatitis of the vulva (34). In addition to more acute conditions, menstrual and intimate care product use may be associated with increased cancer risk of sexual and reproductive organs (e.g., uterine cancer, cervical cancer, ovarian cancer) as well as other gynecologic conditions such as fibroids (39, 41, 68–70). These products could affect chronic health risks through several possible pathways, including inflammation response, microbiota changes, or endocrine disruption (70). Future research should further examine exposure and health consequences of chemicals in menstrual and intimate care products using a combination of *in vitro* and epidemiologic models. Future research should also consider newer, alternative products, such as period underwear, which was reported by several of our study participants in the “other product” category.

### Strengths and limitations

Our analysis has many strengths. Importantly, this work builds upon the environmental injustice of beauty, an intersectional framework that seeks to understand how interlocking systems of power and oppression, and related social identities, shapes beauty norms, product use, chemical exposures, and health across the life course (42, 43). Our data further underscore the importance of the social politics of body odor and personal aroma as upstream drivers of product use, particularly scent-altering products that may contain fragrance chemicals. Our study included a comprehensive examination of nine different menstrual and intimate care products within a diverse cohort. We also examined demographic variations in individual product use as well as analyzed patterns of use across products. Lastly, we included trans and non-binary participants in our study, who have been understudied and under-recognized in environmental and reproductive health research (71).

The study also has some important weaknesses. We relied on cross-sectional surveys that only asked about product use at one time point; product use can change by life stage. We did not ask for information on brands or specific product ingredients, which are critical to evaluating the environmental health risks of reported product use. We lacked adequate statistical power to evaluate multiplicative interactions between race/ethnicity and education, which would better approximate an intersectionality framework. We did not have household income data across both studies. Our only proxy for socioeconomic status was education, which creates a limitation to our socioeconomic analysis, as income may play an important role in determining product use and selection. Our data highlights some important, potential differences in product use by non-binary and transgender populations; however, the size of these subpopulations was small so it is difficult to generalize the findings. Lastly, there were some important differences in the two underlying study populations. While TSS sought to capture product use information among the general, female population in California, FORGE was a clinical epidemiologic study that recruited participants who had gynecologic morbidities (e.g., fibroids, endometriosis) or who were undergoing gynecologic procedures (e.g., hysterectomy). Nonetheless, there were few meaningful differences in product use by study, and adjustment for study in our LCA model did not change associations between product use and our demographic variables of interest. However, because of the unique nature of our study populations, these findings may have limited generalizability, and warrant replication in other study populations.

## Conclusion

We found meaningful differences in menstrual and intimate care product use by race/ethnicity, education, age, and sex/gender, which has important implications for both reproductive and environmental health equity. Importantly, lower educational attainment was consistently associated with greater use of scent-altering menstrual and intimate care products Given the clustered use of scent-altering products by some respondents, which can lead to greater cumulative exposures to fragrance chemicals, regulatory bodies should place greater scrutiny on toxicological evaluation of fragrance chemicals, particularly products used in or near sensitive tissues. In addition to enhanced regulatory actions around product ingredients, there should be greater transparency so that consumers can more easily obtain information on ingredient safety. Future research should examine associations between body odor stigma, product use, and health risks at different intersections of race, class, and gender. The medical community, particularly obstetricians and gynecologists, should be informed on the evolving environmental public health literature on menstrual and intimate care products to provide clearer guidance to their patients on potential health risks. Menstrual activism as a component of feminist politics has increasingly focused on equitable access to menstrual products and promotion of education about menstruation. Our findings suggest that the movement should expand beyond product-focused activism to include examination of root causes of menstrual stigma. Reducing environmental health risks from intimate care and scented menstrual products will require explicitly addressing social norms around menstruation and body odor beginning at an early age. To accomplish this bold task, we will need to shift discourses about menstruation from private to the public sphere, from sanitization and medicalization towards an intersectional lens.

## Data Availability

The original contributions presented in the study are included in the article/**Supplementary Material**, further inquiries can be directed to the corresponding author.

## References

[1] Freidenfelds L. The modern period: Menstruation in twentieth-century America. Baltimore, MD: JHU Press (2009).

[2] MacPhee M. Deodorized culture: anthropology of smell in America. Ariz Anthropol. (1992) 8:89–102.

[3] Upson K Shearston JA Kioumourtzoglou MA. Menstrual products as a source of environmental chemical exposure: a review from the epidemiologic perspective. Curr Environ Health Rep. (2022) 9(1):38–52. 10.1007/s40572-022-00331-1 35302185 PMC9876534

[4] Fortune Business Insights. The global feminine hygiene products market is anticipated to grow from $41.29 billion in 2023 to $62.66 billion by 2030, at a CAGR of 6.1%. Report No.: FBI103530. (2023). p. 160. Available at: https://www.fortunebusinessinsights.com/feminine-hygiene-products-market-103530

[5] Scranton A. Chem fatale: Potential health effects of toxic chemicals in feminine care products. Missoula, MT: Women’s Voices for the Earth. (2013). p. 23. Available at: https://womensvoices.org/menstrual-care-products/chem-fatale-report/#:∼:text=Tampons%20Hazardous%20ingredients%20may%20include,endocrine%20disruption%2C%20and%20allergic%20rash

[6] Jenkins AL Crann SE Money DM O’Doherty KC. “Clean and fresh”: understanding women’s use of vaginal hygiene products. Sex Roles. (2018) 78:697–709. 10.1007/s11199-017-0824-1

[7] Gregory S. Termékteszt: tamponok és egészségügyi betétek. Budapest: Tudatos Vásárlók (2014). Available at: https://tudatosvasarlo.hu/termekteszt-tamponok-es-egeszsegugyi-betetek-0/ (Cited August 30, 2023).

[8] Archer JC Mabry-Smith R Shojaee S Threet J Eckert JJ Litman VE. Dioxin and furan levels found in tampons. J Womens Health. (2005) 14(4):311–5. 10.1089/jwh.2005.14.311 15916504

[9] DeVito MJ Schecter A. Exposure assessment to dioxins from the use of tampons and diapers. Environ Health Perspect. (2002) 110(1):23–8. 10.1289/ehp.0211023 11781161 PMC1240689

[10] Shin JH Ahn YG. Analysis of polychlorinated dibenzo-p-dioxins and dibenzo-furans in sanitary products of women. Text Res J. (2007) 77(8):597–603. 10.1177/0040517507078786

[11] Genet R. OPINION of the French agency for food, environmental and occupational health & safety on the safety of feminine hygiene products. Paris: The French Agency for Food, Environmental and Occupational Health & Safety (2018). Available at: https://www.anses.fr/en/system/files/CONSO2016SA0108EN.pdf

[12] Andrea D. Is there pesticide residue on your tampons? Our independent testing gets specific | naturally savvy. NaturallySavvy.com (2018). Available at: https://naturallysavvy.com/care/is-there-pesticide-residue-on-your-tampons-our-independent-testing-gets-specific/ (Cited August 30, 2023).

[13] Gao CJ Kannan K. Phthalates, bisphenols, parabens, and triclocarban in feminine hygiene products from the United States and their implications for human exposure. Environ Int. (2020) 136:105465. 10.1016/j.envint.2020.105465 31945693

[14] Kuki Á Zelei G Nagy L Nagy T Zsuga M Kéki S. Rapid mapping of various chemicals in personal care and healthcare products by direct analysis in real time mass spectrometry. Talanta. (2019) 192:241–7. 10.1016/j.talanta.2018.09.054 30348385

[15] Singh J Mumford SL Pollack AZ Schisterman EF Weisskopf MG Navas-Acien A Tampon use, environmental chemicals and oxidative stress in the BioCycle study. Environ Health. (2019) 18(1):11. 10.1186/s12940-019-0452-z 30744632 PMC6371574

[16] Lin N Ding N Meza-Wilson E Devasurendra AM Godwin C Park SK Volatile organic compounds in feminine hygiene products sold in the US market: a survey of products and health risks. Environ Int. (2020) 144:105740. 10.1016/j.envint.2020.105740 32866732 PMC7958867

[17] Kim M Park HJ Bae ON Baek SH. Development and uncertainty estimation of cryogenic homogenization and static headspace–gas chromatography–mass spectrometry method for the simultaneous determination of twelve toxic volatiles in disposable menstrual products. Microchem J. (2020) 158:105291. 10.1016/j.microc.2020.105291

[18] Desmedt B Marcelis Q Zhilivoda D Deconinck E. Sensitizing fragrances in absorbent hygiene products. Contact Dermatitis. (2020) 82(5):279–82. 10.1111/cod.13472 31951286

[19] All sanitary pads in Indonesia contain chlorine: YLKI—National—The Jakarta Post. Available at: https://www.thejakartapost.com/news/2015/07/07/all-sanitary-pads-indonesia-contain-chlorine-ylki.html (Cited August 30, 2023).

[20] Office fédéral de la sécurité alimentaire et des affaires vétérinaires (OSAV). *Substances chimiques présentes dans les protections hygiéniques evaluation des risques*. (2016).

[21] Ishii S Katagiri R Kataoka T Wada M Imai S Yamasaki K. Risk assessment study of dioxins in sanitary napkins produced in Japan. Regul Toxicol Pharmacol. (2014) 70(1):357–62. 10.1016/j.yrtph.2014.07.020 25078889

[22] Ding N Batterman S Park SK. Exposure to volatile organic compounds and use of feminine hygiene products among reproductive-aged women in the United States. J Womens Health. (2020) 29(1):65–73. 10.1089/jwh.2019.7785 PMC6998054 31532304

[23] Chow WH Daling JR Weiss NS Moore DE Soderstrom R. Vaginal douching as a potential risk factor for tubal ectopic pregnancy. Am J Obstet Gynecol. (1985) 153(7):727–9. 10.1016/0002-9378(85)90332-1 4073134

[24] Zhang J Thomas AG Leybovich E. Vaginal douching and adverse health effects: a meta-analysis. Am J Public Health. (1997) 87(7):1207–11. 10.2105/AJPH.87.7.1207 9240115 PMC1380899

[25] Klebanoff MA Nansel TR Brotman RM Zhang J Yu KF Schwebke JR Personal hygienic behaviors and bacterial vaginosis. Sex Transm Dis. (2010) 37(2):94. 10.1097/OLQ.0b013e3181bc063c 19823112 PMC2811217

[26] Tang Z Chai M Cheng J Wang Y Huang Q. Occurrence and distribution of phthalates in sanitary napkins from six countries: implications for women’s health. Environ Sci Technol. (2019) 53(23):13919–28. 10.1021/acs.est.9b03838 31694371

[27] Gao CJ Wang F Shen HM Kannan K Guo Y. Feminine hygiene products—a neglected source of phthalate exposure in women. Environ Sci Technol. (2020) 54(2):930–7. 10.1021/acs.est.9b03927 31859481

[28] Park CJ Barakat R Ulanov A Li Z Lin PC Chiu K Sanitary pads and diapers contain higher phthalate contents than those in common commercial plastic products. Reprod Toxicol. (2019) 84:114–21. 10.1016/j.reprotox.2019.01.005 30659930 PMC6504186

[29] Marcelis Q Gatzios A Deconinck E Rogiers V Vanhaecke T Desmedt B. Development and application of a novel method to assess exposure levels of sensitizing and irritating substances leaching from menstrual hygiene products. Emerg Contam. (2021) 7:116–23. 10.1016/j.emcon.2021.02.004

[30] TEST: Menstruationskopper ➡ Test af uønsket kemi i 7 menstruationskopper. Available at: https://taenk.dk/test/kemitest-menstruationskopper (Cited August 31, 2023).

[31] Zhou Y Lin X Xing Y Zhang X Lee HK Huang Z. Per- and polyfluoroalkyl substances in personal hygiene products: the implications for human exposure and emission to the environment. Environ Sci Technol. (2023) 57(23):8484–95. 10.1021/acs.est.2c08912 37262408

[32] Kamazima SR. Vaginal douching: a neglected health risk behavior among women and sexually active adolescent girls in Tanzania? EAS J Psychol Behav Sci. (2023) 5(01):1–9. 10.36349/easjpbs.2023.v05i01.001

[33] Tran TH Steffen JE Clancy KM Bird T Egilman DS. Talc, asbestos, and epidemiology: corporate influence and scientific incognizance. Epidemiology. (2019) 30(6):783–8. 10.1097/EDE.0000000000001091 31469695 PMC6784763

[34] Farage MA. Vulvar susceptibility to contact irritants and allergens: a review. Arch Gynecol Obstet. (2005) 272:167–72. 10.1007/s00404-005-0732-4 15906051

[35] Marcelis Q Gatzios A Deconinck E Rogiers V Desmedt B Vanhaecke T. Quantitative risk assessment of allergens leaching from menstrual hygiene products. Regul Toxicol Pharmacol. (2022) 135:105260. 10.1016/j.yrtph.2022.105260 36067853

[36] Branch F Woodruff TJ Mitro SD Zota AR. Vaginal douching and racial/ethnic disparities in phthalates exposures among reproductive-aged women: national health and nutrition examination survey 2001–2004. Environ Health. (2015) 14:1–8. 10.1186/s12940-015-0043-6 26174070 PMC4502470

[37] Scholes D Daling JR Stergachis A Weiss NS Wang SP Grayston JT. Vaginal douching as a risk factor for acute pelvic inflammatory disease. Obstet Gynecol. (1993) 81(4):601–6. 8459976

[38] Spinillo A Pizzoli G Colonna L Nicola S De Seta F Guaschino S. Epidemiologic characteristics of women with idiopathic recurrent vulvovaginal candidiasis. Obstet Gynecol. (1993) 81(5 (Pt 1)):721–7. 8469460

[39] Gabriel IM Vitonis AF Welch WR Titus L Cramer DW. Douching, talc use, and risk for ovarian cancer and conditions related to genital tract inflammation. Cancer Epidemiol Biomarkers Prev. (2019) 28(11):1835–44. 10.1158/1055-9965.EPI-19-0375 31455671 PMC6825572

[40] Wentzensen N O’Brien KM. Talc, body powder, and ovarian cancer: a summary of the epidemiologic evidence. Gynecol Oncol. (2021) 163(1):199–208. 10.1016/j.ygyno.2021.07.032 34366148 PMC12412271

[41] Ogunsina K Sandler DP Murphy JD Harmon QE D’aloisio AA Baird DD Association of genital talc and douche use in early adolescence or adulthood with uterine fibroids diagnoses. Am J Obstet Gynecol. (2023) 1–10. 10.1016/j.ajog.2023.08.014 PMC10840729 37598998

[42] Zota AR VanNoy BN. Integrating intersectionality into the exposome paradigm: a novel approach to racial inequities in uterine fibroids. Am J Public Health. (2021) 111(1):104–9. 10.2105/AJPH.2020.305979 33211578 PMC7750596

[43] Zota AR Shamasunder B. The environmental injustice of beauty: framing chemical exposures from beauty products as a health disparities concern. Am J Obstet Gynecol. (2017) 217(4):418–e1. 10.1016/j.ajog.2017.07.020 PMC5614862 28822238

[44] Ferranti M. An odor of racism: vaginal deodorants in African-American beauty culture and advertising. Advert Soc Rev. (2011) 11(4). 10.1353/asr.2011.0003

[45] Ferranti M. From birth control to that “fresh feeling”: a historical perspective on feminine hygiene in medicine and media. Women Health. (2010) 49(8):592–607. 10.1080/03630240903496069 20183103

[46] Arbour M Corwin EJ Salsberry P. Douching patterns in women related to socioeconomic and racial/ethnic characteristics. J Obstet Gynecol Neonatal Nurs. (2009) 38(5):577–85. 10.1111/j.1552-6909.2009.01053.x 19883479

[47] Dodson RE Cardona B Zota AR Robinson Flint J Navarro S Shamasunder B. Personal care product use among diverse women in California: taking stock study. J Expo Sci Environ Epidemiol. (2021) 31(3):487–502. 10.1038/s41370-021-00327-3 33958707

[48] Wang VA Chu MT Chie L Gaston SA Jackson CL Newendorp N Acculturation and endocrine disrupting chemical-associated personal care product use among US-based foreign-born Chinese women of reproductive age. J Expo Sci Environ Epidemiol. (2021) 31(2):224–32. 10.1038/s41370-020-00279-0 33235331 PMC7954893

[49] Day JC Newburger EC. The big payoff: educational attainment and synthetic estimates of work-life earnings. special studies. current population reports. (2002).

[50] Hayward MD Hummer RA Sasson I. Trends and group differences in the association between educational attainment and U.S. Adult mortality: implications for understanding education’s causal influence. Soc Sci Med. (2015) 127:8–18. 10.1016/j.socscimed.2014.11.024 25440841 PMC4324094

[51] Diclemente R Young A Painter J Wingood G Rose E Sales J. Prevalence and correlates of recent vaginal douching among African American adolescent females. J Pediatr Adolesc Gynecol. (2012) 25(1):48–53. 10.1016/j.jpag.2011.07.017 22051790 PMC3252400

[52] O’Brien KM Ogunsina K Wentzensen N Sandler DP. Douching and genital talc use: patterns of use and reliability of self-reported exposure. Epidemiology. (2023) 34(3):376–84. 10.1097/EDE.0000000000001589 36652669 PMC10187135

[53] Romo LF Berenson AB. Tampon use in adolescence: differences among European American, African American and Latina women in practices, concerns, and barriers. J Pediatr Adolesc Gynecol. (2012) 25(5):328–33. 10.1016/j.jpag.2012.06.001 22980411

[54] Medina-Perucha L López-Jiménez T Holst AS Jacques-Aviñó C Munrós-Feliu J Martínez-Bueno C Use and perceptions on reusable and non-reusable menstrual products in Spain: a mixed-methods study. PLoS One. (2022) 17(3):e0265646. 10.1371/journal.pone.0265646 35298550 PMC8929555

[55] Van Eijk AM Zulaika G Lenchner M Mason L Sivakami M Nyothach E Menstrual cup use, leakage, acceptability, safety, and availability: a systematic review and meta-analysis. Lancet Public Health. (2019) 4(8):e376–93. 10.1016/S2468-2667(19)30111-2 31324419 PMC6669309

[56] Frank SE. Queering menstruation: trans and non-binary identity and body politics. Sociol Inq. (2020) 90(2):371–404. 10.1111/soin.12355

[57] Stein E Kim S. Flow: the cultural story of menstruation. New York, NY: St. Martin’s Griffin. (2009).

[58] Tickner J Geiser K Baima S. Transitioning the chemical industry: the case for addressing the climate, toxics, and plastics crises. Environ Sci Policy Sustain Dev. (2021) 63(6):4–15. 10.1080/00139157.2021.1979857

[59] Black BC. Oil for living: petroleum and American conspicuous consumption. J Am Hist. (2012) 99(1):40–50. 10.1093/jahist/jas022

[60] Duncan GJ Magnuson K Votruba-Drzal E. Moving beyond correlations in assessing the consequences of poverty. Annu Rev Psychol. (2017) 68:413–34. 10.1146/annurev-psych-010416-044224 27648987 PMC6108837

[61] Aragon A De Los Upton S Flores N Francis D Gunning J Hanebutt R Communicating intimate health. Lanham, MD: Rowman & Littlefield (2021).

[62] Farage MA Miller KW Davis A. Cultural aspects of menstruation and menstrual hygiene in adolescents. Expert Rev Obstet Gynecol. (2011) 6(2):127–39. 10.1586/eog.11.1

[63] Koskenniemi A. Say no to shame, waste, inequality—and leaks! menstrual activism in the market for alternative period products. Fem Media Stud. (2023) 23(1):19–36. 10.1080/14680777.2021.1948885

[64] Crawford BJ Waldman EG. The unconstitutional tampon tax. U Rich Rev. (2018) 53:439.

[65] US Department of Education. National postsecondary student aid study (NPSAS). Washington, DC: Institue of Education Sciences, National Center for Education Statistics (2021).

[66] Markey PM Kurtz JE. Increasing acquaintanceship and complementarity of behavioral styles and personality traits among college roommates. Pers Soc Psychol Bull. (2006) 32(7):907–16. 10.1177/0146167206287129 16738024

[67] Ashford TL. Recounting, rethinking, and reclaiming menstruation. (2003).

[68] Lakshmanan A Chiu YHM Coull BA Just AC Maxwell SL Schwartz J Associations between prenatal traffic-related air pollution exposure and birth weight: modification by sex and maternal pre-pregnancy body mass index. Environ Res. (2015) 137:268–77. 10.1016/j.envres.2014.10.035 25601728 PMC4354711

[69] O’Brien KM Weinberg CR D’Aloisio AA Moore KR Sandler DP. The association between douching, genital talc use, and the risk of prevalent and incident cervical cancer. Sci Rep. (2021) 11(1):14836. 10.1038/s41598-021-94447-3 34290340 PMC8295379

[70] O’Brien KM D’Aloisio AA Shi M Murphy JD Sandler DP Weinberg CR. Perineal talc use, douching, and the risk of uterine cancer. Epidemiology. (2019) 30(6):845–52. 10.1097/EDE.0000000000001078 31584892 PMC6779343

[71] Bucher ML Anderson FL Lai Y Dicent J Miller GW Zota AR. Exposomics as a tool to investigate differences in health and disease by sex and gender. Exposome. (2023) 3(1):osad003. 10.1093/exposome/osad003 37122372 PMC10125831

