# Supplementary material for: Examining differences in menstrual and intimate care product use by race/ethnicity and education among menstruating individuals
Source: Front Reprod Health. 2023 Dec 6;5:1286920. doi: 10.3389/frph.2023.1286920 (PMC10731462; doi:10.3389/frph.2023.1286920)
Supplement: Supplementary file 1 [file Datasheet1.docx]

***Supplementary Material***

**Examining differences in menstrual and intimate care product use by race/ethnicity and education among menstruating individuals**

Ami R. Zota^1^, Elissia T. Franklin^2^, Emily B. Weaver^1^, Bhavna Shamasunder^3^, Astrid Williams^4^, Eva L. Siegel^1^, Robin E. Dodson^2^

^1^Department of Environmental Health Sciences, Mailman School of Public Health, Columbia University, New York, NY, USA

^2^Silent Spring Institute, Newton, MA, USA

^3^Departments of Urban and Environmental Policy and Public Health, Occidental College, Los Angeles, CA, USA

^4^Black Women for Wellness, Los Angeles, CA USA

***Correspondence:**

Ami R. Zota, ScD, MS

arz2124@cumc.columbia.edu

***Supplemental Figure 1.*** Concordance in product use. Higher concordance (phi estimate) indicated by darker shades.


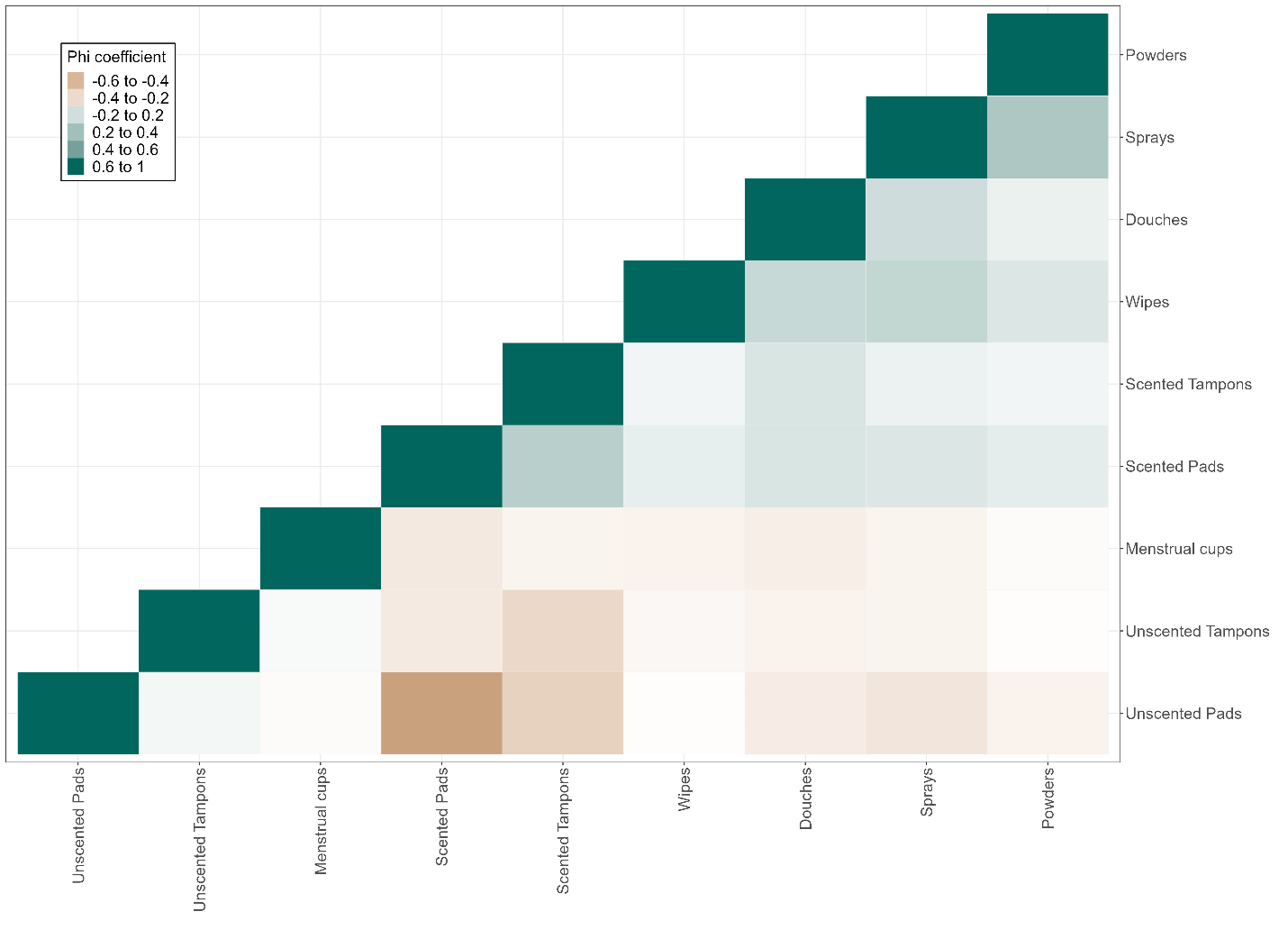


***Supplemental Table 1.*** Relative Risks (95% Cis) from mutually adjusted log-binomial models excluding 18-24 year olds. Statistically significant differences (p<0.05) from the referent group are indicated by an asterisk and **bold**. Referent groups: Age—25-34 years, Race/Ethnicity—Black, Education—Bachelor’s or more, Study—TSS (N=460)

| term | Unscented Pads | Unscented Tampons | Menstrual cups | Scented Pads | Scented Tampons | Wipes | Douches | Sprays | Powders |
| --- | --- | --- | --- | --- | --- | --- | --- | --- | --- |
| **Age** |  |  |  |  |  |  |  |  |  |
| 25 - 34 | REF | REF | REF | REF | REF | REF | REF | REF | REF |
| 35 - 44 | 0.95 (0.84, 1.1) | 1.1 (0.85, 1.3) | **0.52 (0.29, 0.95)*** | 1.7 (0.83, 3.5) | 1.6 (0.51, 4.9) | 0.96 (0.67, 1.4) | 0.97 (0.49, 1.9) | 0.92 (0.47, 1.8) | 2.1 (0.46, 9.7) |
| 45 - 54 | 0.94 (0.8, 1.1) | 1.1 (0.83, 1.4) | **0.15 (0.045, 0.48)*** | 1.8 (0.81, 4) | 1.8 (0.54, 6.2) | 0.99 (0.65, 1.5) | 1.5 (0.7, 3) | 0.34 (0.11, 1) | 2.1 (0.4, 11) |
| **Race** |  |  |  |  |  |  |  |  |  |
| Black | REF | REF | REF | REF | REF | REF | REF | REF | REF |
| Latinx | 1.1 (0.99, 1.3) | 1.1 (0.87, 1.5) | 1.1 (0.47, 2.5) | 1.1 (0.55, 2.2) | 0.63 (0.2, 1.9) | **0.58 (0.39, 0.86)*** | 0.52 (0.26, 1) | 0.54 (0.26, 1.2) | **0.2 (0.039, 1)*** |
| White | 0.9 (0.74, 1.1) | **1.7 (1.3, 2.1)*** | 2.1 (0.99, 4.5) | 1.5 (0.7, 3.2) | 1.6 (0.51, 4.8) | **0.53 (0.3, 0.93)*** | **0.24 (0.059, 0.97)*** | 0.78 (0.29, 2.1) | 1.4 (0.45, 4.6) |
| Some other  identity | 1.1 (0.9, 1.3) | 1.3 (0.91, 1.7) | 2.3 (0.98, 5.2) | 1.3 (0.53, 3.3) | 1.3 (0.34, 4.8) | 0.61 (0.35, 1.1) | 0.55 (0.2, 1.5) | 0.54 (0.17, 1.8) | -- |
| **Education** |  |  |  |  |  |  |  |  |  |
| High school  or less | **0.76 (0.64, 0.92)*** | **0.5 (0.34, 0.73)*** | **0.11 (0.015, 0.8)*** | **3.7 (2, 7.2)*** | **4.6 (1.6, 13)*** | **1.5 (1, 2.3)*** | **4.2 (2.1, 8.2)*** | **5.1 (2.2, 11)*** | **4.8 (1.1, 22)*** |
| Some college/technical school/associate degree | 0.89 (0.78, 1) | **0.78 (0.62, 0.99)*** | **0.41 (0.17, 0.95)*** | **2.4 (1.2, 4.5)*** | **3 (1.1, 8.3)*** | **1.6 (1.2, 2.3)*** | **2.5 (1.3, 4.9)*** | **3.4 (1.5, 7.7)*** | **4.8 (1.3, 18)*** |
| Bachelor’s or  more | REF | REF | REF | REF | REF | REF | REF | REF | REF |
| **Study** |  |  |  |  |  |  |  |  |  |
| TSS | REF | REF | REF | REF | REF | REF | REF | REF | REF |
| FORGE | 1.1 (0.97, 1.3) | 1.1 (0.87, 1.3) | 1.8 (0.91, 3.6) | 1.2 (0.62, 2.2) | 0.93 (0.34, 2.5) | 0.9 (0.62, 1.3) | 1 (0.53, 1.9) | 0.77 (0.34, 1.7) | 0.37 (0.099, 1.4) |

***Supplemental Table 2.*** Demographic differences by latent class excluding 18-24 year olds. Adjusted RRs (95% CI) are for relative risk of having membership in the scent-altering product class. Significant differences (p<0.05) are indicated by an asterisk and **bold**.

|  | Scent Altering Product Class | |  |
| --- | --- | --- | --- |
| **Characteristic** | **Yes (N=64)** | **No (N=408)** | Adjusted RR (95% CI) |
| **Age** |  |  |  |
| 25 - 34 | 13 (20.3%) | 131 (32.1%) | REF |
| 35 - 44 | 34 (53.1%) | 181 (44.4%) | 1.4 (0.74, 2.5) |
| 45 - 54 | 17 (26.6%) | 96 (23.5%) | 1.5 (0.76, 3) |
| **Race/ethnicity**^[[1]](#footnote-1)^ |  |  |  |
| Black | 25 (39.7%) | 168 (41.8%) | REF |
| Latinx | 21 (33.3%) | 134 (33.3%) | 0.84 (0.45, 1.6) |
| White | 11 (17.5%) | 54 (13.4%) | 1.8 (0.95, 3.3) |
| Some other identity | 6 (9.5%) | 46 (11.4%) | 0.97 (0.4, 2.3) |
| **Education**^[[2]](#footnote-2)^ |  |  |  |
| High school or less | 23 (37.1%) | 65 (16.0%) | **4.4 (2.4, 7.9)*** |
| Some college/technical school/associate degree | 21 (33.9%) | 102 (25.2%) | **2.5 (1.4, 4.6)*** |
| Bachelor’s or more | 18 (29.0%) | 238 (58.8%) | REF |
| **Sex/gender** |  |  |  |
| Female | 63 (98.4%) | 394 (96.6%) | NA |
| Transgender | 1 (1.6%) | 7 (1.7%) | NA |
| Non-binary | 0 (0.0%) | 7 (1.7%) | NA |
| **Study** |  |  |  |
| TSS | 46 (71.9%) | 303 (74.3%) | REF |
| FORGE | 18 (28.1%) | 105 (25.7%) | 0.93 (0.52, 1.7) |

1. 7 participants did not report race/ethnicity [↑](#footnote-ref-1)
2. 5 participants did not report education [↑](#footnote-ref-2)
